# Mixed Large Cell Neuroendocrine Carcinoma of the Ovary: Report of a Rare Case

**DOI:** 10.1155/2020/8896070

**Published:** 2020-11-25

**Authors:** Ilias Galanis, Georgios Floros, Maria Sotiria Bompoula, Christophoros Theodoropoulos

**Affiliations:** ^1^2nd Department of Surgery, Evaggelismos General Hospital, Ipsilantou 45-47, Athens 106 76, Greece; ^2^3rd Department of Obstetrics and Gynecology, Attikon general hospital, Rimini 1, Haidari 124 62, Greece

## Abstract

LCNC (large cell neuroendocrine carcinomas) of the ovary is a rare aggressive tumor entity of the genital tract. Its pathogenesis, origin, and prognosis have not been fully elucidated, since there are a limited number of cases reported in literature. We report a case of an 80-year-old patient, who presented with a growing abdominal mass, which turned out to be a mixed LCNC/epithelial neoplasm. Although this type of tumor is rare, the continuous development of pathologoanatomy and immunohistochemistry contributes to our better knowledge and comprehension of these neoplasms.

## 1. Introduction

Ovarian NETs (neuroendocrine tumors) accounting for only 0.5% of all NETs and 0.1% of all ovarian cancers [1] are associated with a conventional surface epithelial tumor or a teratoma. Pure ovarian NETs (47%) are usually larger in size and present higher rates of metastases and carcinoid syndrome [2–4].

NETs of the ovary may appear as relatively benign ovarian carcinoids (with their four subtypes: insular, trabecular, strumal, and mucinous) or as LCNC (non-small cell carcinoma), as in our case, and small cell carcinomas of the pulmonary type, both associated with poor patient prognosis [5]. Here, we report a case of a neuroendocrine carcinoma of a non-small cell type with an admixture of serous carcinoma.

## 2. Case

**Figure 1:**
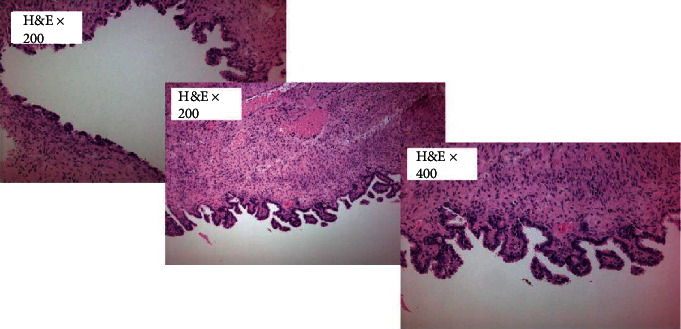
Serous borderline tumor/atypical proliferative serous tumor.

**Figure 2:**
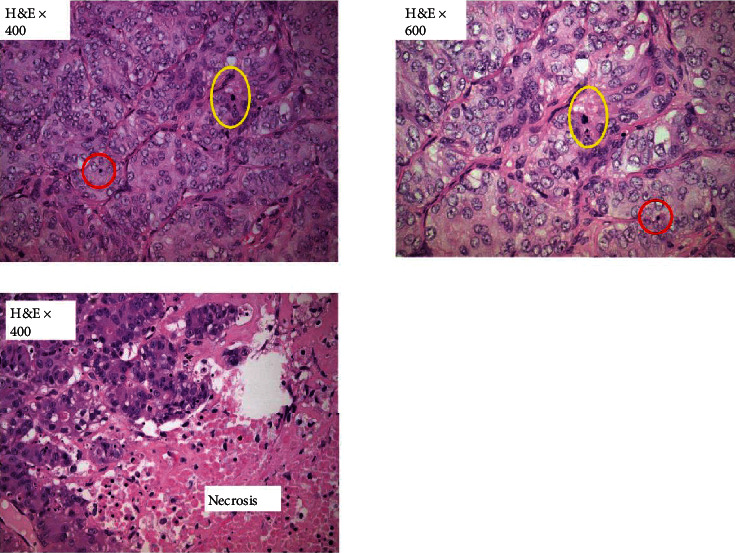
High-grade large cell neuroendocrine carcinoma. High mitotic (yellow circle) and apoptotic (red circle) activity. Extended necrotic areas.

**Figure 3:**
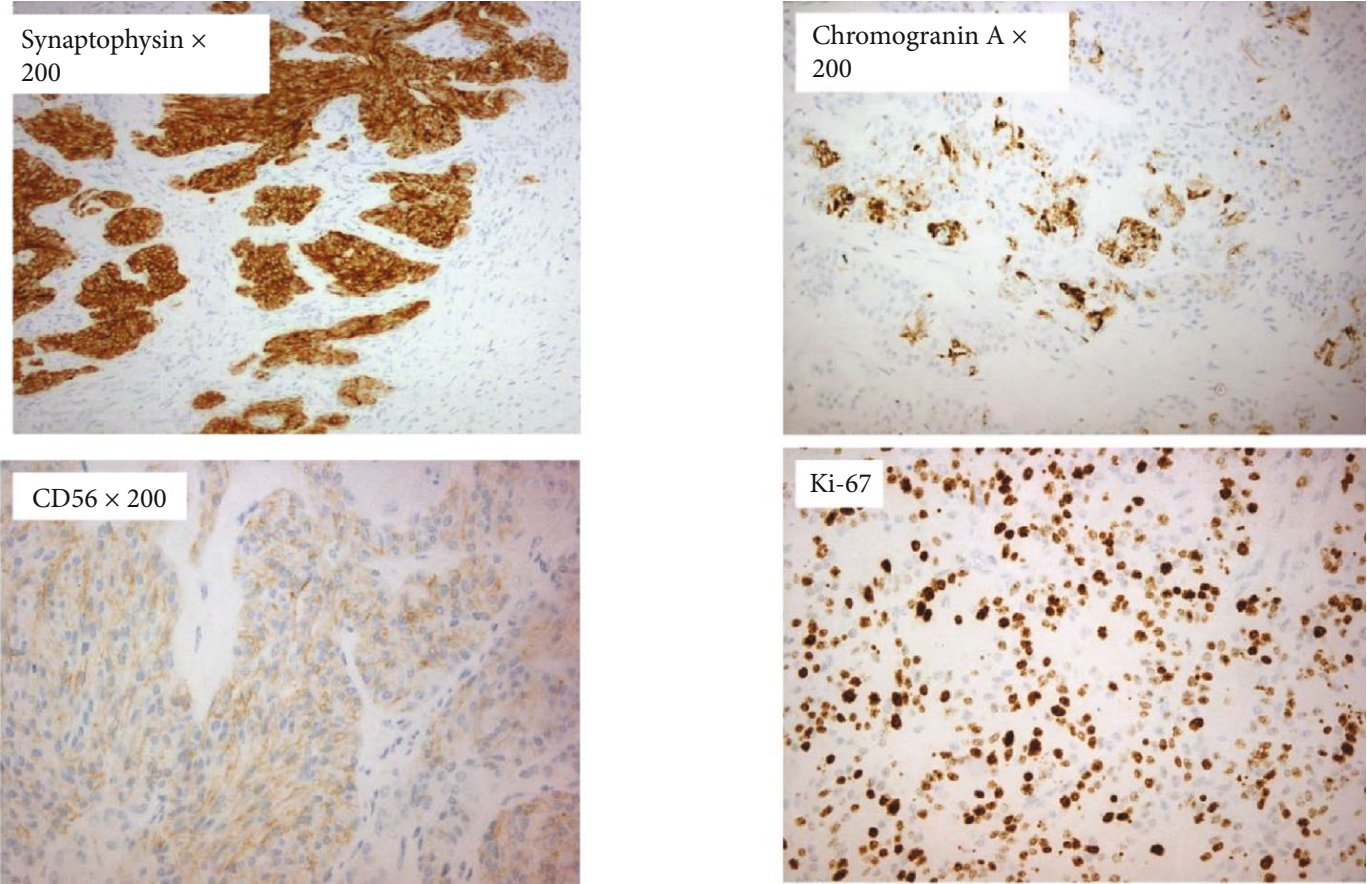
Positive neuroendocrine markers (chromogranin, synaptophysin, and CD56). High Ki-67 proliferation index.

An 80-year-old woman, with a history of coronary artery disease, hypertension, and chronic obstructive pulmonary disease, was admitted to our surgical department with abdominal pain and distension. A palpable mass in the right inguinal area at the physical examination was investigated further with a CT (computed tomography) scan of the abdomen. A big cystic tumor deriving from the right ovary mandated surgical exploration. A large cyst of 13 × 10 × 5 cm was found intraoperatively on the right ovary. Considering the patient's age and her comorbidities from the cardiovascular and respiratory system, minimal surgical intervention was performed to avoid the complications of long-time anesthesia. A right salpingooophorectomy was performed. Frozen sections of the contralateral ovary were, also, performed during surgery, which proved to be negative. There were no signs of ascites or peritoneal metastasis. The pathologoanatomical report showed a mixed ovarian neoplasm with a cystic and a solid component. The cystic component contained a serous borderline/atypical proliferative tumor (Figure 1) while the solid one appeared to be a high-grade LCNC, which was developed under the serous neoplasm on the cystic wall and comprised 40% of the tumor (stage pT1a/AJCC/2017). Microscopically, the LCNC were characterized by the presence of islands, sheets, and trabeculae with little intervening stroma, cell necrosis, and high mitotic activity (Figure 2). Immunohistochemically, chromogranin and CD56 (cluster of differentiation 56) were partially while synaptophysin ubiquitously expressed in the neuroendocrine cells (Figure 3). CDX-2 (caudal-type homeobox transcription factor 2) and TTF-1 (thyroid transcription factor 1) were negative, as well as inhibin and calretinin. Ki-67 proliferation index was positive in up to 80% of the cells. Mitotic rate was 17 mitosis/10 HPFs (high-power fields). Subsequently, the patient underwent adjuvant chemotherapy, six cycles of carboplatin, and paclitaxel every three weeks. She has been on follow-up by CT scan every six months and has no evidence of disease recurrence, almost two years later.

## 3. Discussion

Neuroendocrine cells can be divided into two systems, cells that constitute glands (pituitary, thyroid, parathyroid, ganglia, thymus, and adrenal medulla) and diffusely distributed dispersed cells that constitute a disseminated system which is referred to as the diffuse neuroendocrine system (DNES). Embryologically, these systems are different, because the former is derived from ectodermal tissue and the latter is from endodermal tissue. Epithelial tissue can be, also, derived from either the ectoderm or the endoderm. The epithelial tissue derived from the endoderm includes the epithelial lining of the digestive tract, except at the open ends, and the epithelial lining of all hollow structures formed as outpockets in the digestive tract [5]. This common origin of epithelial and neuroendocrine cells explains the frequent association of neuroendocrine cancer with an epithelial component. According to bibliographic research, ovarian LCNC, although rare, always coexist with surface epithelial tumors, most frequently with serous borderline tumor.

LCNC are higher grade and more aggressive tumors than carcinoids. They present organoid growth pattern, as well as cellular heterogeneity. The tumor cells are commonly medium-sized to large and express moderate atypia, high mitotic activity, and extensive cell necrosis [6]. Patients exhibit symptoms of abdominal pain and distension and can complain about abdominal discomfort or even a palpable mass [7].

The most important tool in our armamentarium for the diagnosis of NETs is immunohistochemistry. The markers most frequently used for diagnosis of ovarian NETs include synaptophysin, chromogranin, and CD56 [8]. A highly positive Ki-67 is indicative of a poor prognosis. These tumors are negative for epithelial membrane antigen (EMA), estrogen, and progesterone receptors and sex-cord stromal markers such as inhibin and calretinin [1]. Immunohistochemistry has become an essential examination for the identification and differential diagnosis of neuroendocrine carcinomas with other primary or metastatic tumors. Over the last decade, the diagnostic accuracy of specific organ or tumor immunomarkers has improved significantly. For instance, TTF-1 is a nuclear transcription factor that promotes embryogenic pulmonary and thyroid differentiation and is expressed by most lung and thyroid neoplasms. CDX-2 is a homeobox transcription factor expressed in the nuclei of intestinal epithelial cells and serves as a biomarker for gastrointestinal tumor differentiation, especially colorectal [3]. These biomarkers demonstrate great potential utility in suggesting the primary site of poorly differentiated neuroendocrine carcinomas [9]. In our case, both TTF-1 and CDX-2 were negative. CT and magnetic resonance imaging (MRI) usually show *nonspecific findings* in *LCNC* cases. CA-125 levels, regularly used to monitor epithelial ovarian cancer, are not specific to clinical courses for LCNC. 5-Hydroxyindole acetic acid (5-HIAA), in contrast, is a sensitive marker of neuroendocrine components in ovarian tumors [2].

Surgical resection of the tumor is the cornerstone of therapy [8]. There is no standard systemic treatment regimen. Some treatment options for advanced or metastatic ovarian carcinoids include streptozocin, 5-fluorouracil, capecitabine, and cisplatin. Somatostatin analogues may be used, especially if symptoms of carcinoid syndrome are present. Finally, due to altered expression in the mTOR signaling pathway, which has been observed in NETs, mTOR (mammalian target of rapamycin) inhibitors constitute a promising treatment option [1, 10].

## 4. Conclusion

Although rare tumors, LCNC are being increasingly reported in the last few years, possibly thanks to the increased availability of immunohistochemical diagnostic tools and awareness. General consensus on their standard therapy has not yet been established, but early diagnosis is crucial because of the biological aggressiveness of these neoplasms.

## Data Availability

The data that support the findings of this case report are available from the corresponding author, I. Galanis, upon reasonable request.
